# Exploring why animal health practices are (not) adopted among smallholders in low and middle-income countries: a realist framework and scoping review protocol

**DOI:** 10.3389/fvets.2022.915487

**Published:** 2022-07-29

**Authors:** Arata Hidano, Hannah Holt, Anna Durrance-Bagale, Mehroosh Tak, James W. Rudge

**Affiliations:** ^1^Communicable Disease Policy Research Group, Department of Global Health and Development, Faculty of Public Health and Policy, London School of Hygiene and Tropical Medicine, London, United kingdom; ^2^Veterinary Epidemiology, Economics and Public Health Group, Royal Veterinary College, London, United Kingdom; ^3^Faculty of Public Health, Mahidol University, Bangkok, Thailand

**Keywords:** livestock, smallholders, LMICs, intervention, adoption, uptake, One Health, realist synthesis

## Abstract

**Background:**

Improving livestock health is considered critical to address poverty, malnutrition and food insecurity in low- and middle-income countries (LMICs). Modifications of livestock management practices is also increasingly recognized as an important strategy to mitigate global threats such as climate change and novel disease emergence. Smallholders are, however, under various constraints which prohibit them from altering health practices for livestock and little is known about how the adoption of these practices may be promoted. The proposed scoping review aims to systematically map evidence around “what practices are (not) adopted by smallholders under what circumstances, how and why?.”

**Method and analysis:**

We conducted initial scoping searches to broadly define types of animal health practices relevant for smallholders in LMICs and formulated search terms. A scoping review protocol was designed and registered. A systematic literature search will be conducted using electronic databases including CAB Abstract, Scopus, MEDLINE, EMBASE, and Web of Science Core Collection. Gray literature will be searched from AGRIS and Standards for Supporting Agricultural Livelihoods in Emergencies. Articles in English, pertaining to the animal health practices considered highly relevant will be considered eligible for inclusion. Articles will be screened at two stages by two independent reviewers; screening of titles, abstracts, and keywords, followed by full-article screening. The first reviewer will review 100% of the articles at both stages. The second reviewer will review a random sample of 20% of the articles at both stages. Any disagreements will be resolved using inputs from the third reviewer. A thematic analysis will be conducted to catalog contexts and mechanisms for adoption and discussed under a realist framework.

**Discussion:**

Understanding of the mechanisms underlying the adoption of animal health practices by livestock smallholders in LMICs is crucial for successful implementation of interventions including those which are based on a One Health approach. This review will identify the extent of this knowledge across disciplines and inform future research priorities for the design of effective and feasible interventions which can contribute toward Sustainable Development Goal 2.

**Registration:**

This protocol is registered within the Open Science Framework (https://doi.org/10.17605/OSF.IO/FUQAX).

## Background

### Livestock interventions in low and middle-income countries

A report from the World Health Organization in 2013 states that one of the critical challenges faced by the global health community is the failure to effectively implement interventions in the real world (1). In order to deliver intended health outcomes, interventions that are empirically proven effective under certain conditions still need to be accepted, adopted, and feasible to sustain among targeted individuals and communities (2). Interventions involving livestock are no exception.

Livestock farming contributes to people's livelihood in low- and middle-income countries (LMICs) through multiple pathways including income generation, food security, and providing assets for agricultural products and culture. Studies have shown that livestock production in LMICs is far from optimal and could yield three to four-fold higher outputs (3, 4), rendering livestock development programs an attractive tool among governments, NGOs, and other development actors for reducing poverty and malnutritional (5). Despite this importance, the literature provides limited evidence of the impacts, especially long-term ones, delivered by livestock interventions (6, 7). A recent scoping review of feed interventions for livestock keepers in LMICs highlighted that there is a disproportionately low number of studies that evaluated the adoption of interventions among target populations (8).

Adoption is one of the first key steps of interventions. Many studies in high-income countries have shown that the adoption of new technologies and livestock management practices among farmers is a non-linear and complex process (9–11). Furthermore, smallholders in LMICs often face systematic constraints (12–14). For instance, smallholders have limited market access and bargaining power, providing little incentive for investment to improve the health of livestock and the quality of livestock products (15). This is further reinforced by frequent outbreaks of livestock diseases with high mortality, such as African Swine Fever and Highly Pathogenic Avian Influenza. These disease not only incur production losses but often force smallholders to sell their livestock at an unfavorable price (16, 17). Livestock development policies in LMICs often provide subsidies for industrial farming systems, creating market forces that do not favor small-scale producers (18). Many smallholders cannot intensify their production scale due to the vast resources required, and even when it is possible such intensification may not be profitable for smallholders (12, 18). This “vicious cycle” of mutually reinforcing constraints makes it extremely challenging for smallholders to alter their behaviors and adopt interventions targeting only single constraints.

### Leveraging One Health paradigm

This existing effort to tackle the “avicious cycle” may be leveraged through growing recognition of the role of livestock interventions in addressing key global threats of the 21^st^ Century. For instance, integration of livestock into farming with appropriate grazing and manure management practices, could help maintain soil fertility and thus sustainable and resilient food productions under climate change (19, 20). The SARS-Cov-2 pandemic re-emphasizes the paramount importance of prevention and early detection of emerging and re-emerging diseases at the human-animal interface, particularly in LMICs which are considered hot-spots for the emergence and dissemination of novel pathogens (21, 22). The widespread overuse and misuse of antibiotics and anthelmintics in livestock contributes to the global rise in antimicrobial resistance, threatening the availability of effective treatments to manage both human and animal disease (23). Meanwhile, the suboptimal coverage of vaccines that can prevent important infectious diseases has been observed across the world in both sectors (24, 25). These examples highlight the shared problems between human and animal sectors, and synergies generated from a One Health approach that encompasses the health of humans, animals and the environment (26). One Health interventions could potentially break the “vicious cycle” among smallholders by simultaneously targeting multiple systematic constraints, if effective interventions were designed, and then accepted and adopted by communities. This requires understanding not only of the systematic constraints mentioned above, but also of causal mechanisms through which these constraints work together to prohibit behavioral changes among smallholders.

### Limitations of “barriers and facilitators” knowledge synthesis

In public health literature, there has been an increase in systematic reviews that synthesize research on barriers and facilitators for various outcomes such as intervention uptake, access to health care, and adherence to prescribed treatment. While such knowledge is attractive to decision-makers and can provide a useful description of underlying problems, these systematic reviews have been criticized for several reasons (27). The main criticism pertains to their reductionist approach, which oversimplifies complex and dynamic human decision-making processes (28). For instance, this approach frames each barrier (and facilitator) as independent, which in turn can lead to assumptions that removal of an identified barrier will help achieve a desired outcome, whereas in reality it may have unintended consequences, such as creating another barrier or crushing other facilitators (29). Furthermore, a recent systematic review of “barrier and facilitator systematic reviews” identified various issues in this type of systematic review (27). For instance, these reviews may be biased toward reporting barriers and facilitators that are uncontroversial or of *a priori* interest of primary researchers. Another issue identified was that these reviews struggle to make sense of differential impacts when factors identified as barriers in one study were considered facilitators in another study. This issue seems to relate to the lack of definition of barriers and facilitators, and the context-dependent effect of these factors. One potential solution to overcome these issues is to explicitly describe and evaluate the context in which factors operate using approaches such as realist synthesis.

### Realist synthesis

Realism is a methodological orientation or a philosophy of science (30, 31). A realist approach acknowledges the existence of an external social reality, which we may not directly observe, and this reality influences human behavior. A realist synthesis thus inquires “What works for whom under what circumstances, how and why?” (32), rather than “Is the intervention effective or not?.” This method was developed in response to the need of overcoming the difficulty of applying high quality evidence generated by methods with high internal validity such as randomized control trails (RCTs) to different contexts. Unlike the conventional view of intervention evaluation which focuses on the “pure” effect size of given interventions, a realist perspective interprets the interaction between context and mechanism that results in the outcome; mechanism is a generative force of outcome and mechanism is triggered by context. This interaction is referred to as context-mechanism-outcome (CMO) configuration. Realism acknowledges that the effect of a specific mechanism is dependent on other mechanisms as well as contexts and, therefore, that an effort to understand the “pure” effect of an intervention by controlling for context may limit our ability to understand “how, when and for whom the intervention will be effective” (33). By theorizing CMO configuration(s) behind an intervention, a realist synthesis aims to provide an explanation about the context in which, or population groups for which, an intervention will or will not work. Box 1 and Table 1 provide brief descriptions of each terminology and examples of CMO configurations, respectively.

Box 1 **Realist synthesis terminology**

***Context:*** The various features, factors, and conditions that surround an intervention. Non-exhaustive examples include [33];

*Situational context*: setting in which the program occurs (e.g., hospital, smallholders), characteristics of the program (e.g., funding availability, experience of program staff) and participants (e.g., gender, economic situation);

*Social context*: culture, social norms, historical stability, policy and legislation, other past and on-going interventions/programs;

*Geographical context*: country, geographical features (e.g., access);

*Other relevant context*: relationship between community and program implementer

Although there can be a large number of contexts for each population and intervention, the key in a realist synthesis is to identify contexts that trigger and/or modify mechanisms of interest, which generate outcomes [31].

***Mechanism:*** A realist philosophy defines mechanisms as “causal processes (forces) that generate outcomes” [31]. Mechanisms operate at both the individual and social/structural levels. Mechanisms are context sensitive, meaning that particular mechanisms are triggered through an interaction between specific contexts and interventions. That is, a mechanism is not inherent to an intervention or program activity, but is a function of the participants and the context. The same intervention can trigger different mechanisms for different participants even within one location [33]. Realism assumes that the intervention or program activity works in the following steps [33]. First these activities change resources or opportunities available for participants, altering their decision-making; this process is understood as changing the context. Second, the new context triggers mechanism(s), which generates outcome(s). Mechanism may be invisible and not a variable, but an account that can be studied from observable data. The objective of realist synthesis is thus to infer this account, and refine this to develop a theory that explains how, why and when intervention works for whom. This theory needs to be specific enough to generate testable hypothesis but general enough to be applied to other interventions in other contexts. This characteristic is referred to as “middle-range theory” [32].

***Outcome***: Outcomes can be both intended and unintended. There can be multiple outcomes, each of which can work as a stepping-stone of a large program. For each outcome, there can be multiple sets of CMO configurations [33].

**Table 1:** Examples of context-mechanism-outcome configurations.

**Intervention**	**Contexts of participants and intervention**	**Mechanisms to be triggered**	**Outcomes achieved or expected to occur**
Training of community health workers (CHWs) (34)	1. Intervention was implemented in urban communities who were poor and had an unmet need. 2. Intervention was linked to local public health care services and implemented by locally trusted agencies. 3. Intervention involved CHWs from the beneficiary community and those trusted and seen as role models by the community. 4. Training targeted specific situations, supplemented by practice sessions and on-job mentoring.	Sense of self efficacy among CHWs; CHWs gained enactive mastery of the tasks and confidence in solving problems through skill building and practice sessions.	Positive intended outcomes such as promoting breast feeding and diarrhea prevention.
Providing knowledge regarding appropriate antimicrobial prescription for doctors-in-training (35)	Doctors-in-training are under hierarchical dynamics that influence prescribing decisions.	Fear of criticisms and of individual responsibility for patients deteriorating.	Interventions that provide only knowledge or skills are less effective because the hierarchical dynamics hinder trainees to apply their knowledge.
A campaign targeting responsible ownership for dog owners to improve population-level physical activity (36) ^a^	Urban communities where people are afraid of uncontrolled dogs.	Sense of safety and connectedness in the community; more dogs under control reduces peoples' fear of dogs. Dog-owners picking up dog litter facilitates sense of living in ‘a good neighborhood'.	Increased physical activity in the population.

^a^*CMO configuration was not fully established in this study as this was a scoping review*.

### This scoping study: toward understanding of “what works for livestock smallholders under what circumstances, how and why?”

A realist synthesis is a time and resource demanding process, which involves identifying initial rough theory [which may be composed of theory of change and theory of action (37)] followed by an iterative process of refining and testing the theory against the literature. For this reason, uncertainty in the amount of adequate evidence in the literature can discourage efforts to employ this type of synthesis (38). Given the suggestion that many RCTs report limited information regarding intervention contexts and poorly explained mechanisms (34), searching and locating relevant information across disciplines is essential; however, to the authors' knowledge, such studies have not been conducted for smallholders' livestock health practices. This scoping review aims to fill this gap by systematically mapping of information regarding CMO configurations for livestock health practices for both infectious and non-infectious diseases.

## Methods

If this protocol needs amendments, the date as well as the description of change of each amendment will be presented in the scoping review.

### Objective

The objective of this scoping review is to explore the availability and nature of information regarding contexts and mechanisms for the adoption of animal health practices relevant to prevention, management, control and treatment of livestock infectious and non-infectious diseases among smallholders in low- and middle-income countries (LMICs).

### Search strategies

A systematic search, selection, and mapping of published literature will be used in this scoping review to answer the research question. This review protocol has been registered in the Open Science Framework (registration https://doi.org/10.17605/OSF.IO/FUQAX). The study protocol is reported in accordance with Preferred Reporting Items for Systematic reviews and Meta-Analyses extension for Scoping Reviews (PRISMA-ScR) (39) and developed using the framework previously proposed (40, 41).

### Identifying the research question

The research question is; what is the extent and nature of evidence reported regarding the context and mechanisms behind adoption and non-adoption of animal health practices among smallholders in LMICs? This research question was formulated based on the SPIDER tool (42, 43) while ensuring the search includes quantitative, qualitative and mixed-method studies. The SPIDER tool was developed to overcome limitations of the PICO tool, which is not necessarily suitable for identifying qualitative studies (42).

### Defining animal health practices

Given there are no universal definitions of “animal health practices,” we first defined types of practices/behaviors that are relevant to prevention, management, control and treatment of livestock diseases among smallholders in LMICs. To this end, we conducted initial scoping searches to identify types of animal health practices that are studied in both animal health and social science literature, which identified 12 themes/concepts considered highly relevant in this study as shown in Table 2 (see Supplementary Tables 1–3 in Supplementary File 1 for the full details). Theme “feed management” was excluded from this study because there is a recent scoping review on this topic (8) and the inclusion of this theme would significantly broaden the study scope. It should be noted that these themes/concepts are not mutually exclusive; for instance, “human-animal relationships” and “wellbeing and stress management” are inter-related. Also other relevant themes such as intensified farming are not explicitly listed because such themes can be considered combinations of multiple themes that are included in this study (e.g., intensified farming requires a change in the housing system and vaccination/antimicrobial use).

**Table 2:** Themes/concepts for animal health practices that were considered of high relevance in this study.

	**Theme/concept**	**Description**	**Exemplar behaviors/practices included in the theme**
1	Biocontainment	Practices that may influence how infectious diseases spread among animals on farm	Quarantine new/sick animals, removal of sick animals, pre-emptive culling, keep multiple animal species on farm, use a fallow period between animal introductions, all-in-all-out
2	Cleaning and disinfection of livestock environment	Practices that influence the sanitation of livestock environment	Cleaning and disinfection of animal pens/houses, provide a hygienic environment for livestock
3	Animal introduction	Selecting the source of animals that are introduced to farms/backyard	Purchase animals only from neighbors, outside of the village, vendors or specific sources
4	Feed and water safety	Practices that may influence the contamination/hygiene status of feed and water for livestock	Disinfect drinking water for livestock, use natural water sources, feed uncooked food, feed animal's bodies or organs, feed commercial feed that may contain antibiotics, clean food/water containers/troughs
5	Fomites and mechanical vector	Management of farm materials and devices that may influence how infectious disease spreads	Share farm materials with neighbors, change/disinfect boots and footwear, wash feeding utensils, disinfect/clean/dry equipment, restrict visitors, use of artificial insemination, biosecurity practices
6	Health monitoring and seeking	Monitor, treat and report sick and/or abnormal livestock	Test animal health status, report animal sickness/death, keep record of herd health, use animal health services
7	Human-animal relationship	Practices or statuses between human and livestock that may influence how smallholders deal with and care livestock	Build a friendship and mutual trust with livestock
8	Medicine, supplement and chemical substance	Use of medicines, supplements and chemical substances that may influence animal health status	Use of feed additives and antimicrobial substances, vaccination, use of antiviral/anti-parasite drugs, prophylaxis, use of traditional therapy, use of anti-inflammatory drugs, use of dipping, hoof care, herd health
9	Pest and vector control	Practices that may influence the extent of the contact between livestock and pests and biological vectors	Use of insect net, use insecticide, vermin/pest/vector control
10	Animal management	General animal management and husbandry practices that may affect animal health status	Raising animals indoor/enclosures, tethering animals, scavenging/free grazing, not sharing animal housing with neighbors/individual housing, share breeding male, use of local/exotic breeds
11	Waste management	Management of livestock waste and carcass	Disposal of carcass, aborted and birthing biological materials, management of manure and biogas
12	Well-being and stress management	Practices that may influence the level of stress on livestock	Management of animals giving birth, conditions of floor and bedding, wound management and injuries of animals, care of animals

### Identifying relevant studies

Primary research articles will be systematically searched from the following electronic databases; CAB Abstracts, Scopus, MEDLINE, EMBASE, and Web of Science Core Collection. Gray literature will be searched from AGRIS and Standards for Supporting Agricultural Livelihoods in Emergencies. Reference lists of eligible articles will be hand-searched to identify any relevant articles that are not retrieved by the database search. The search strategy was developed through consultation with an information specialist, followed by further team discussion. Search terms were developed for each theme relevant to animal health by identifying keywords and subject headings, where appropriate, which encapsulate the concept of each theme and behavior included in the theme. Search terms and subject headings for each theme used can be found in Supplementary Tables 4–7. These search terms are used in combination with the key concepts in the research question; LMICs, smallholder, and livestock. See Supplementary Files 2–5 for the full search strategies used in each database.

### Study selection

We will use the following inclusion and exclusion criteria and studies should meet all inclusion criteria and none of the exclusion criteria to be eligible for inclusion.

#### Inclusion criteria

1. Study population includes livestock keepers that have at least one of the following animal types to produce livestock products; cattle, domesticated buffalo, sheep, goat, pig, horse, or poultry;
2. Primary empirical research or review;
3. Study describes the study population's (non)adoption and/or views of animal health practices of interest, defined above;
4. Abstract reports smallholders' livestock practice or indicates that information of practices/behaviors were collected as part of the study (this criterion will be applied in the title and abstract screening);
5. Full text available in English.

#### Exclusion criteria

1. Study does not take place in LMICs or does not review farmer behavior in LMICs;
2. Study does not report any information on farm practices/behaviors;
3. Study only looks at farmer behaviors/practices outside the farm (e.g., slaughterhouse and market).
4. Abstract does not mention ‘livestock' or at least one of livestock species of interest defined above.
5. Study only looks at livestock feeding/feed management (e.g., growth performance due to specific feed);
6. Experimental studies that are not carried out at smallholders;
7. Book chapter;
8. Conference abstract.

Search results obtained from all sources listed will be exported to Covidence (https://www.covidence.org/), a web-based software specialized for systematic review. Duplicate citations will be removed using the software function. Articles will be screened at two stages using inclusion/exclusion criteria by two independent reviewers; screening of titles, abstracts, and keywords, followed by full-article screening. The first reviewer will screen 100% of the articles at both stages. The second reviewer will screen a random sample of 20% of the articles at both stages. Reasons for exclusion will be documented. Disagreement in the screening results at the first stage between two reviewers will be identified and discussed. Where disagreements cannot be resolved, the third reviewer will be involved to refine the study selection and data extraction standard. This process will be repeated at the second screening process. A flow chart showing the detailed selection process will be provided.

### Charting the data

A data-charting form will be developed by the first reviewer and then refined through an iterative process of piloting the form and further team discussion. The chart will be developed in Microsoft Excel and include the following variables; authors, year of publication, study location(s), study population, sample size, study aim, target species (including humans), theme/concept and name of behavior of interest, uptake percentage (if any), factors influencing the behavior of interest reported by the authors, study design (including whether it involved an intervention, and if any, duration and assumption of the intervention), type(s) of disease investigated or targeted (if any), and other important results. Furthermore, detailed information of contexts will be extracted (see Box 1). Mechanism(s) will be extracted if they were identified or discussed by the authors of each eligible study.

### Quality appraisal

We expect substantial heterogeneity in study design of the included articles, which are likely to include observational and intervention studies, and present evidence from a range of qualitative and quantitative domains. Furthermore, this scoping review draws on a realist synthesis framework, which rejects the methodological hierarchy and considers that “bad research may yield good evidence” (38, 44). In a realist synthesis, quality appraisal is conducted based on two criteria; relevance and rigor. These criteria are applied to particular data that is relevant for the synthesis rather than to a whole document or article (32). For these reasons, we will not remove any studies based on the quality or characteristic of articles. Instead, we use the following four variables to represent the amount and general quality of information; key limitations of the study, richness of context descriptions [using 1-5 scale for assessing data richness developed by (45)], richness of reasoning to explain mechanisms for adoption/non-adoption (Not stated–Limited–Moderate –Rich). For the “richness of reasoning” variable, the following examples describe (but are not limited to) what fall into each category: Not stated-no explanation about (non)adoption; Limited - explanations given that do not consider the study context; Moderate-explanations consider the study context but causal mechanisms for (non)adoption are not discussed; Rich-causal mechanisms for (non)adoption in relation to the study context discussed in-depth.

### Collating, summarizing and reporting the results

We will first conduct descriptive analysis to describe the scope and nature of studies included, stratified by study types. A table that summarizes characteristics of studies in each study type will be created. As described earlier, mere cataloging of barriers and facilitators oversimplifies the reality and is unlikely to generate information that can directly improve interventions; nevertheless, this analysis is useful for understanding the nature of research within a given topic, in particular where such a catalog does not exist yet. Therefore, barriers and facilitators reported by the authors will be categorized based on a socio-ecological framework, which groups factors into individual, inter-personal, community and macro-policy level (46). We will then qualitatively assess any differential impacts of identified barriers and facilitators across studies. Finally, we evaluate the extent and characteristics of evidence regarding contexts, mechanisms and their interactions. Narrative will be structured by sub-headings based on important themes that emerge during the analysis.

## Discussion

Continuing efforts to improve holistic health through livestock development and the One Health paradigm can have an synergetic impact on poverty, gender inequity, and malnutrition (13), to name a few. These together have the potential to break the “vicious cycle” faced by smallholders in LMICs. One of the critical knowledge gaps is the mechanism for the adoption, or non-adoption, of practices associated with livestock health. A recent study highlighted the detailed account on how specific diseases are prioritized and livestock management practices are developed under a highly contextualized environment (47). Through synthesizing such evidence, we aim to fill the knowledge gap by not only mapping barriers and facilitators reported across disciplines, but also turning our attention toward better causal understanding of adoption in relation to contexts.

This scoping review may have some limitations. We will not conduct a full synthesis to develop and refine theories for better behavioral adoptions. The outcome of interest is also limited to adoption rather than final intervention outcomes (e.g., disease reduction). These are inevitable because the amount and nature of evidence is uncertain at this stage. Despite these limitations, the study results will be useful for assisting decision-makers and researchers to formulate and/or refine hypotheses regarding why livestock health interventions are adopted or not in a given context. This study will also identify knowledge gaps and future research priorities for the design of effective and feasible livestock interventions for poverty reduction, biosecurity and food security.

Configurative evidence synthesis, which aim to interpret and arrange information [e.g., realist synthesis, meta-narrative review (48)], is gaining its popularity in the health literature as an alternative method to traditional systematic review (49). These two approaches complement each other; traditional systematic review and meta-analysis can produce a more precise understanding of a phenomenon of interest (e.g., treatment efficacy) and configurative synthesis can explore the difference between studies. Configurative approach has a promising place in animal and veterinary domains, in which the number of randomized controlled trials is limited (7, 50), while qualitative studies on farmers, health of non-humans, and human-animal interactions continue to increase (51, 52). Indeed, a call for realist framework is not new in the One Health research arena (53). We hope this scoping review will serve as a catalyst for attracting more interest in evidence integration across disciplines and generating knowledge on causal mechanisms for “what works, when and why?.”

## Data availability statement

The original contributions presented in the study are included in the article/Supplementary material, further inquiries can be directed to the corresponding author.

## Author contributions

AH: conception and design of the work, the acquisition, analysis, interpretation of data, and drafting the manuscript. HH, AD-B, MT, and JR: design of the work and drafting the manuscript. All authors have approved the submitted version, and agreed to be accountable for their contributions as well as the integrity of the manuscript.

## Funding

AH, HH, and JR were supported by funding from the U.S. Department of Defense, Defense Threat Reduction Agency (PigFluCam+ project; HDTRA1-18-1-0051). The content of the information does not necessarily reflect the position or the policy of the federal government, and no official endorsement should be inferred.

## Conflict of interest

The authors declare that the research was conducted in the absence of any commercial or financial relationships that could be construed as a potential conflict of interest.

## Publisher's note

All claims expressed in this article are solely those of the authors and do not necessarily represent those of their affiliated organizations, or those of the publisher, the editors and the reviewers. Any product that may be evaluated in this article, or claim that may be made by its manufacturer, is not guaranteed or endorsed by the publisher.

## Supplementary material

Supplementary Tables Data extracted and used for defining animal health practices among smallholders.
